# Roux-en-Y Gastric Bypass after Laparoscopic Sleeve Gastrectomy Failure: Could the Number of Previous Operations Influence the Outcome?

**DOI:** 10.3390/jcm13010293

**Published:** 2024-01-04

**Authors:** Federico Sista, Sergio Carandina, Antoine Soprani, Emmanuel Rivkine, Laura Montana, Fabiana Fiasca, Sonia Cappelli, Antonella Grasso, Marius Nedelcu, Irene Tucceri Cimini, Marco Clementi

**Affiliations:** 1 Hepatic Pancreatic and Biliary Surgical Unit, San Salvatore Hospital, Department of Biothecnological and Applied Clinical Sciences, University of L’Aquila, 67100 L’Aquila, Italy; 2 ELSAN, Clinique Saint Michel, Centre de Chirurgie de l’Obésité (CCO), 83100 Toulon, France; 3 Department of Digestive and Bariatric Surgery, Clinica Madonna della Salute, 45014 Porto Viro, Italy; 4 Clinique Geoffroy-Saint Hilaire, Générale de Santé (GDS), Department of Digestive and Bariatric Surgery, 75005 Paris, France; antoinesoprani@hotmail.com; 5 Department of Digestive and Bariatric Surgery, Centre Hospitalier Universitaire de Martinique, 97261 Fort-de-France, France; 6 Department of Digestive and Metabolic Surgery, Groupe Hospitalier Diaconesses Croix Saint-Simon, 75012 Paris, France; 7 Public Health Unit, Department of Biotechnological and Applied Clinical Sciences, University of L’Aquila, 67100 L’Aquila, Italy; 8 Department of Surgery, IRCCS Regina Elena National Cancer Institute, 00144 Rome, Italy; 9 General Surgical Unit, San Salvatore Hospital, Department of Biotechnological and Applied Clinical Sciences, University of L’Aquila, 67100 L’Aquila, Italyirene.tuccericimini@graduate.univaq.it (I.T.C.)

**Keywords:** obesity, sleeve gastrectomy, multiple restrictive procedures, Roux-en-Y gastric bypass, bariatric surgery

## Abstract

After a failed laparoscopic adjustable gastric band (LAGB), laparoscopic sleeve gastrectomy (LSG) has been proposed as revisional surgery. Those patients that receive a second restrictive procedure fall into a small subgroup of patients with more than one restrictive procedure (MRP). If also the second restrictive procedure fails, the correct surgical strategy is a challenge for the surgeon. Roux-en-Y gastric bypass (RYGB) may be an option but there is no evidence in the literature on whether the procedure is effective in treating failures after MRP. This study aims to evaluate the influence of the previous number of restrictive interventions (MRP vs single LSG) in the results of RYGB as revisional surgery. We have retrospectively analyzed patients who underwent conversion from laparoscopic sleeve gastrectomy (LSG), or from multiple restrictive procedures (MRP), to RYGB for weight regain (WR) or insufficient weight loss (IWL) between 2009 and 2019. The number of patients analyzed was 69 with conversion to RYGB after LSG and 44 after MRP. The reduction of excess weight (%TWL) at 3, 6, 12, 24 RYGB postoperative months was respectively of 11.03%, 16.39%, 21.43%, and 24.22% in the MRP group, and of 10.97%, 16.4%, 21.22%, and 22.71% in the LSG group. No significant difference was found in %TWL terms after RYGB for the MRP group and the LSG group with an overall %TWL, which was 11.00 ± 6.03, 16.40 ± 8.08, 21.30 ± 9.43, and 23.30 ± 9.91 respectively at 3, 6, 12, and 24 months. The linear regression model highlighted a positive relationship between the %EWL post-bypass at 24 months and the time elapsed only between the LSG and RYGB in the MRP group patients (*p* < 0.001). RYGB has proved to be a reliable technique with good results in terms of weight loss after failed bariatric surgery both in patients who previously underwent MRP and in those who underwent exclusively LSG. RYGB showed better results in patients who experienced WR than in those who had IWL from previous techniques.

## 1. Introduction

Restrictive bariatric surgery offers excellent results in the treatment of obesity and associated metabolic comorbidities [1]. However, obesity being a chronic disease, it requires treatments that allow for lasting results with low long-term complication rates. For these reasons, all bariatric procedures have a certain percentage of failures related to insufficient weight loss, weight recovery, poor control of comorbidities, or the onset of new problems such as dysphagia and gastroesophageal reflux disease [2,3,4,5]. In total, up to 25% of patients undergoing bariatric surgery require a revisional surgery which is more complex, and with a consequent high rate of postoperative complications [6,7,8,9]. Revisional surgery has proven to be an effective and safe option for these patients, but there is still considerable heterogeneity in the choice of the right intervention after first-line surgery [10,11]. Historically, a laparoscopic adjustable gastric band (LAGB) is the bariatric procedure most likely to be subject to revisional surgery due to its high rate of failures. LSG is used as a rescue procedure in a significant number of these patients [12,13]. In recent years, the SG has been the preferred restrictive intervention for treating obesity. Even with this procedure, there is a chance of failure that requires revision surgery. The management of these patients can either include a second LSG (re-LSG) or conversion to more malabsorptive techniques such RYBP, OAGB, or SADI [14,15].

In revisional surgery after restrictive surgery, such as laparoscopic sleeve gastrectomy (LSG) or laparoscopic adjustable gastric banding (LAGB), the patients that receive a second restrictive procedure by LSG or re-LSG fall into a small subgroup of patients with more than one restrictive procedure (MRP). 

If the second restrictive procedure also fails, the correct surgical strategy is a challenge for the surgeon. Roux-en-Y gastric bypass (RYGB) may be an option. There is no evidence in the literature as to whether RYGB is also effective in patients who have previously undergone more than one restrictive procedure (MRP) such as LAGB followed by LSG.

The aim of this study is to compare, in a multicenter retrospective analysis, the results of revision surgery with RYGB in patients who have previously undergone MRP (LAGB after one or two LSG) with those of patients who have previously undergone LSG only. 

## 2. Materials and Methods

### 2.1. Study Design and Investigations

Three hundred and seventy-four (374) patients who underwent RYGB surgery after LSG and after MRP between 2010 and 2019 were analyzed in a retrospective study involving two French university hospitals, three French private centers, and one Italian private center. The indication for revision was weight loss failure after LSG regardless of whether it was insufficient weight loss (IWL) or weight regain after achieving a satisfactory result (WR) [9]. We considered WR as regaining weight to achieve a BMI > 35, or %EWL > 25% with respect to the minimum weight after the previous procedures, in accordance with the literature data [16]. IWL was considered as when the patient achieved a %EWL inferior to 50% two years after LSG, according to Reinhold’s criteria [17]. All patients converted for complications following restrictive techniques or severe gastroesophageal reflux (GERD) were excluded from the study. In addition, all patients who had not completed a follow-up of at least 2 years were excluded. 

The preoperative workup before the conversion included an upper gastrointestinal series, gastroscopy, and nutritional, cardiologic, endocrinologic, and anesthesiologic evaluations. All patients with endoscopically detected *Helicobacter pylori* infection underwent a bacterium eradication treatment before surgery.

Patients were divided into two groups (LSG group and MRP group) based on their bariatric surgical history. We included patients in the MRP group who had undergone LASGB followed by LSG prior to RYGB. In the LSG group, we included patients who had only undergone a gastrectomy sleeve prior to RYGB.

Demographic characteristics, body mass index (BMI), %TWL and %EWL at 3, 6, 12, and 24 months were then evaluated. 

Postoperative complications were classified based on the Clavien–Dindo classification (CD) and the time of onset (early, during the first postoperative month; or late, after the first postoperative month) [18].

In order to study the effects of previous interventions on the results of conversion to RYGB, we considered %TWL in the two groups as a primary outcome at least 2 years after the revisional surgery, and we also analyzed the correlation with the time elapsed between the LSG surgery and RYGB.

As a secondary outcome, we analyzed the correlation, within the two groups, between %TWL at 24 months and %TWL after the previous procedures, focusing on the cases who had a weight regain (WR) and those who had exclusively an insufficient weight loss (IWL).

The mean value obtained at 12 months (MV-12) after RYGB was identified as the cut-off to determine the failed results at 24 months of post-MRP malabsorptive surgery [19,20,21].

Hypertension was considered in case of systolic blood pressure ≥140 and/or diastolic blood pressure ≥90 mmHg, or use of antihypertensive drug therapy, while a patient was considered suffering from type 2 diabetes when fasting plasma glucose was ≥126 mg/dL or using anti-diabetic drugs with or without insulin therapy. Severe obstructive sleep apnea was considered when a high apnea/hypopnea index and/or the need for continuous positive airway pressure during sleep were present. The remission of any comorbidity was defined as the patient no longer needing drug therapy and showing normal blood pressure and lab values. Concerning OSA, remission was considered in the case of cessation of continuous positive airway pressure machine usage. For diabetes, remission was defined as normal fasting glucose without medication for 1 year, and a glycosylated hemoglobin (A1C) of <6.5%. Improvement was defined as changing from insulin to oral anti-diabetic drugs, lowering the dose or number of drugs needed, or an improvement in A1C with the same treatment. 

The ethical approval to conduct this study was granted by the Institutional Review Board (IRB) of the study promoter center (protocol code 19141 and date of 14 February 2023 IRB University of L’Aquila). Due to the retrospective nature of the study, it waived the need for informed consent for the use of deidentified patient data. The study was also approved by the ethical board committees of the different French private centers; clinical trial registration: TCTR20221010001.

### 2.2. Surgical Technique and Follow-Up

The laparoscopic RYGB was performed on the patient with legs apart in the Anti-Trendelenburg position using the 5-port technique (5–12 mm, @Ethicon endosurgery, Cincinnati, OH, USA). The stomach was dissected approximately at the level of the second vessel of the lesser gastric curvature, in order to create a gastric pouch of approximately 30 cc in volume.

In the case of dilated sleeves, a re-sleeve was performed using a probe around 38 Fr. The biliary loop was prepared approximately 80 cm from the Treitz ligament, and the alimentary loop was performed at a length of 150 cm, and subsequently anastomosed in antecolic manner to the gastric stump.

The end-lateral gastro-jejunal anastomosis was performed with a linear stapler (45 mm, blue-gold reloads, Echelon, @Ethicon endosurgery, Cincinnati, OH, USA). The service orifices were closed with a resorbable double-layer manual suture. The later-lateral jejuno-jejunal anastomosis was made with a linear stapler (60 mm, blue reloads, Echelon, @Ethicon endosurgery, Cincinnati, OH, USA).

Following RYGB, the patients were re-fed on the second postoperative day after a double test with the methylene blue test and an upper gastrointestinal series.

The patients were discharged between the 3rd and the 5th postoperative day after restarting eating.

Upon discharge, patients were sent home with proton pump inhibitor (PPI) therapy, multivitamin complexes, and a special diet for gastric bypass. A follow-up was performed respectively at 3, 6, 12, 18, and 24 postoperative months.

### 2.3. Statistical Analysis

A descriptive analysis of the characteristics of the sample was carried out, stratifying by type of pre-RYGB intervention (MRP and LSG). Discrete and nominal variables (sex and pre-RYGB condition) were described in terms of absolute frequencies and percentages, the difference between the two groups being analyzed with Fisher’s exact test. The continuous variables (age, BMI before LSG, MRP/LSG–RYGB timing, BMI before RYGB, %EWL before RYGB, %TWL and %EWL after RYGB at 3, 6, 12, and 14 months) were reported as means and standard deviations, and the differences between the two groups were analyzed using the Student’s *t*-test for independent samples. The slope analysis of the regression line was used to study the trend of %EWL at 3, 6, 12, and 24 months in the two groups. The relationship of %EWL at 24 months with sleeve duration and %EWL after the penultimate surgery in MRP and LSG groups was analyzed using a linear regression model. Using a univariate logistic regression model, the dependent variable is represented by the success/failure of the RYGB intervention. It is defined as “success” in the situation with cases showing %EWL 24 months > %EWL 12 months, and “failure” in the opposite condition (%EWL 24 months ≤ %EWL 12 months). The factors associated with a higher probability of bypass surgery failure were analyzed considering age, sex, pre-sleeve BMI, sleeve duration, pre-bypass BMI, and type of pre-bypass intervention as explanatory variables. The associations were reported as odds ratio (OR) and 95% confidence intervals. The tests used were bidirectional, and the significance level was set at 5%. The data were processed using the statistical package STATA/IC15.0.

## 3. Results

**Figure 1:**
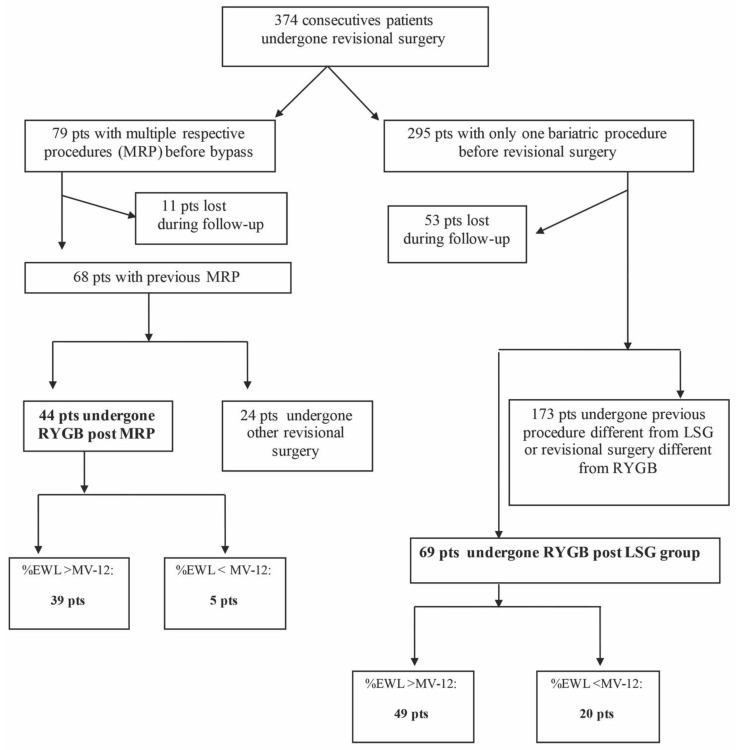
Flow chart. LSG: laparoscopic sleeve gastrectomy, MRP: multiple restrictive procedures, RYGB: Roux-en-Y bypass.

From the original database of patients submitted to revisional bariatric surgery between 2010 and 2019, 113 patients were selected and included in the study: 44 patients in the RYGB group post-MRP, and 69 in the RYGB group post-LSG (Figure 1).

As shown in the flow chart (Figure 1), 79 patients were initially included in the MRP group but 11 patients were lost to follow-up. In the remaining 68 patients, 24 patients had other surgery and were excluded from the analysis. Forty-four patients who underwent RYGB post-MRP revision surgery were included in the study. One anastomosis gastric bypass (OAGB) was performed as an alternative to RYGB in 24 (100%) cases.

The number of patients who underwent revision surgery after LSG failure was 295, but 52 were lost to follow-up. In the final 242 patients, 69 had RYGB and have been included in the study, while 173 had other revision surgery and have been excluded from the analysis. The interventions performed as an alternative to RYGB were: 7 re-sleeve (S-LSG), 3 (1.7%) biliopancreatic diversion (BPD), 5 (2.9%) single-anastomosis duodenoscopic duodenal bypass (SA-DI-S), and 158 (91.3%) OAGB.

The analysis of the general characteristics of the two groups shows overlap in terms of age, sex, BMI pre-sleeve and pre-bypass, time elapsed between sleeve and RYGB, and obesity-related comorbidities (Table 1). The incidence of arterial hypertension in the MRP and LSG groups was 22.7% and 17.4%, respectively. Six patients in the MRP group (13.6%) and 5 (7.2%) in the LSG group had T2D, while 14 (31.8%) in the MRP group and 18 (26.1%) in the LSG group had severe SAOS. 

In the MRP group, the timing between the positioning of the LAGB and its removal was 65.8 months (25–190). The incidence of LAGB removal and LSG in a single procedure (35 patients) was significantly higher than that of the two-stage procedure (LAGB removal following LSG in a second operation, 9 patients).

The average of the interval between the last restrictive surgery and RYGB was 49.6 months, with no significant differences between the two groups. After the last restrictive surgery, the overall %EWL was 7.22 ± 52.32 with significant differences between the two groups (*p* < 0.001), respectively 22.40 ± 73.72 for the MRP group and 25.67 ± 14.56 for the LSG groups. IWL before RYGB was significantly higher in the LSG group (100%) than in the MPR group (68.18%) (*p* < 0.001) while WR was significantly higher in the MRP group (14%) than in the LSG group (0%) (*p* < 0.001).

There was no mortality and no conversion to open surgery in both groups. The overall rate of early complications was 8.8% (10 patients), with no statistically significant difference between the two groups (*p* = 0.5). Four patients experienced postoperative bleeding (two in MRP group and two in the LSG group; *p* = 0.6) and needed a laparoscopy for hemostasis (CD III). One patient in the MRP group presented a leak of the gastro-jejunal anastomosis at postoperative day 2 that was treated with endoscopic stent positioning and antibiotics (2.3%) (CDIII). Two patients in the MRP group experienced bleeding from gastro-jejunal anastomosis (4.5%), and one patient in the LSG group from jejuno-jejunal anastomosis (1.4%). In all those cases, bleeding was managed conservatively (CDII). Two patients in the LSG group (2.9%) experienced postoperative pneumonia and needed IV antibiotic administration (CDII). Late complications were recorded in three patients (2.6%): a port site hernia in the MRP group and two bowel obstructions in the LSG group. All those patients underwent a reoperation in order to fix the problem (CD III).

No significant difference was found in %TWL terms after RYGB for the MRP group and the LSG group, with an overall %TWL that was 11.00 ± 6.03, 16.40 ± 8.08, 21.30 ± 9.43, and 23.30 ± 9.91, respectively, at 3, 6, 12, and 24 months (Table 1).

**Figure 2:**
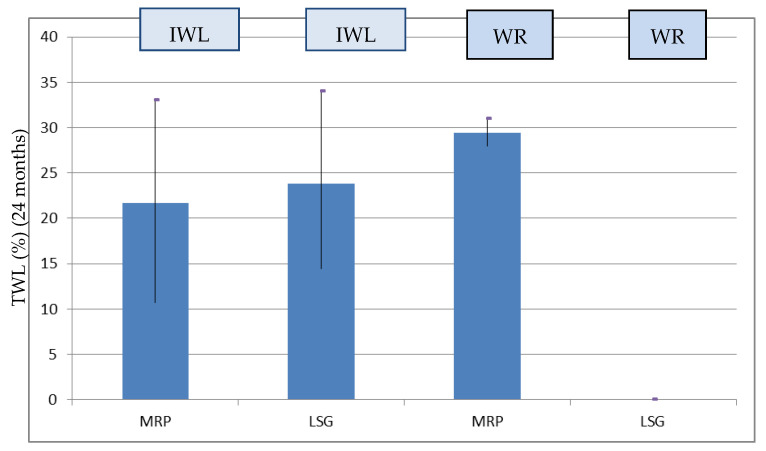
%TWL at 24 months after RYGB stratified for IWL and WR in the groups.

In the patients who experienced IWL after LSG (99 patients), the %TWL at 24 months was higher in the LSG group (69 patients) compared to the MPR group (30 patients) (Table 1), but there was no statistically significant difference (22.71 ± 1.08 vs 21.62 ± 2.25, *p* = 0.621). All the patients who experienced WR (14 patients) had previously undergone MPR (%TWL 24 months: 29.80 ± 6.02) (Figure 2).

**Figure 3:**
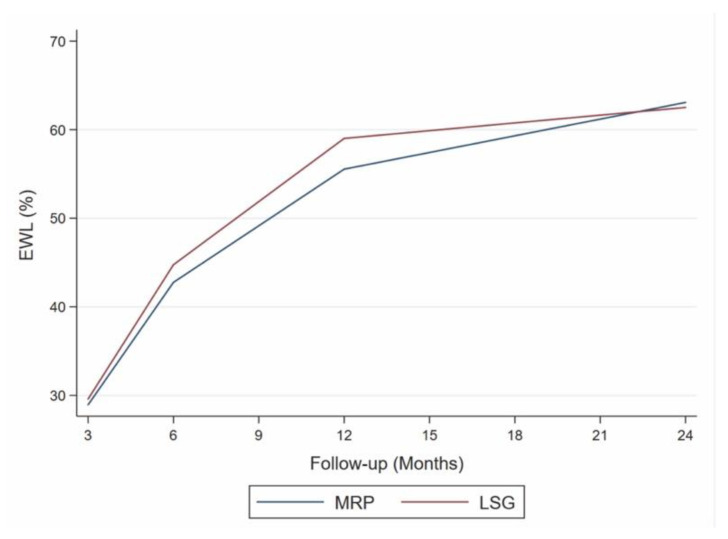
Excess weight loss (EWL) at various follow-up intervals. LSG: laparoscopic sleeve gastrectomy, MRP: multiple restrictive procedure trend test. MRP: β coefficient = 1.48; 95% CI = −0.49–3.45, *p* = 0.084; LSG: β coefficient = 1.40; 95% CI = −1.07–3.87, *p* = 0.135.

The mean value of %TWL obtained at 12 months (MV-12), chosen as the cut-off, was 21.3% with a non-significant overlap in the trends of the two groups (Figure 3).

In the MRP group, the patients who experienced %TWL at 24 months > 21.3% (MV-12) had timing between the last restrictive surgery and RYGB shorter than the LSG group, although there was no statistical significance (48.76 ± 28.91 vs 52.64 ± 29.15, *p* = 0.556). 

The linear regression model highlighted a positive relationship between the %EWL post-bypass at 24 months and the time elapsed between the LSG and RYGB in the MRP group patients (*p* < 0.001), thus showing an average increase of 0.5% in EWL for each month of time elapsed between the two procedures. In the case of the LSG group patients, there was no linear relationship (*p* = 0.404). There was also a significant negative linear relationship (*p* = 0.031) between the %EWL post-bypass at 24 months and the %EWL after the penultimate surgery in patients undergoing MRP. The %EWL post-bypass at 24 months decreased by an average of 0.12 as the %EWL increased by one unit after the penultimate intervention (Table 2).

Table 3 shows the logistical analysis of the factors associated with the probability of failure of RYBP.

The %EWL < 57.6% at 24 months was significantly greater for patients in the LSG group than for the MRP group (OR 3.18, 95% CI 1.10–9.25, *p* = 0.033). No difference was found for the time elapsed between the penultimate surgery and RYGB for the two groups.

Comorbidities had an overall postoperative resolution and/or improvement rate of 80% in the MRP group and 77.1% in the LSG group, without statistically significant differences between the two groups (*p* = 0.7). Specifically, OSAS had complete resolution with the discontinuation of positive pressure therapy in 87.5% of cases (28/32). Diabetes mellitus improved in 63.6% of cases (7/11), leading to a reduction in medication, while in the other patients remained stable (4/11). Hypertension resolved completely in nine (41%) patient with discontinuation of therapy, seven patients reduced the dosage of antihypertensives (31.8%), and it remained stable in six cases (23.7%). The various comorbidities’ responses to surgical treatment did not show significant differences between the two groups (Table 4).

## 4. Discussion

Although the surgical strategies for the treatment of obesity are all effective with excellent %EWL at 24 months, some authors have shown a weight recidivism varying from 5.7% to 20% after restrictive procedures such as LSG [3,4]. A revisional surgery is often mandatory for these patients. 

However, the choice for the best revisional surgical strategy after the failure of a restrictive procedure is complex due to the multifactorial nature of the failure, and frequently this determines disagreement in the literature, not allowing the standardization of guidelines [3,6,8,22,23,24,25,26].

In fact, the revisional surgery must take into account the multiple reasons that lead to this choice. In the case of LAGB, the failure is often attributable to poor compliance of the patient with the band and to complications of the device itself, and in the case of LSG to the unsatisfactory weight reduction, or to the presence of severe GERD [6,22,24]. 

Our mostly descriptive study aims to evaluate the impact of previous number of restrictive procedures (LASB + LSG vs. LSG alone) on weight control (%TWL and %EWL) on outcomes of RYGB as revisional surgery, excluding patients who required revision due to a complication after the restrictive procedure adopted.

In the recent past, laparoscopic adjustable gastric band (LAGB) has been the bariatric most likely subject to revisional surgery due to its high rate of failures [11]. LSG has been used as a rescue procedure in a significant number of these patients when failure occurs due to poor weight control [10]. In practice, it has been shown that LSG also has a failure rate of about 30% during a long follow-up [27,28]. This fact shows that, according to recent data on revisional surgery, LSG is the most frequently revised procedure with an estimated revision rate of 20%, while Roux-en-Y gastric bypass is the most common revision procedure [11].

When LSG does not provide good weight control or allows weight loss recidivism, a shift toward a rescue malabsorptive procedure appears to be widely accepted. In a recent consensus statement on revisional bariatric surgery, experts agreed that RYGB, one-anastomosis gastric bypass (OAGB) and single anastomosis duodeno-ileal bypass (SADI-S) were acceptable rescue bariatric surgery options after failed restrictive surgery [26]. All these types of techniques have a strong malabsorptive component and have demonstrated excellent results in terms of weight loss but with a higher long-term risk of metabolic complications and malnutrition. Unfortunately, there is currently a lack of randomized studies of high scientific value focusing on which of these techniques can provide a good balance between the recovery of weight loss after LSG and the patient’s quality of life.

Although RYGB guarantees good results and low complications in patients with LSG failure, all the studies analyzed patients with revision surgery after a single restrictive surgery (LSG or LAGB). The mixed of malabsorptive and restrictive surgery resulting from the two procedures offers results in terms of %EWL ranging from 48% to 82.1% [8,21,29,30]. In the present study, the results recorded are consistent with the literature data, with an average %EWL of 62%. However, our results were obtained on patients who had previously undergone two failed restrictive procedures (LASB followed by LSG).

The present study did not show significant differences at 24 months between patients previously undergoing LSG or MRP in terms of %TWL and WR rate after RYGB, despite the fact that the two groups experienced different weight loss results after the previous surgery. In other words, RYGB seems to give excellent results as a revisional technique even in patients with a history of multiple restrictive procedures.

However, in this subset of patients, other options seem to provide equivalent or even better results. In our previous study, we compared OAGB and RYGB for the treatment of patients who underwent more than one restrictive procedure [8]. We found that the results of weight loss after several restrictive procedures appear to show a better response after OAGB compared to RYGB after 2 years of follow-up.

The good results reported in our study are also confirmed by the improvement/resolution of patients’ comorbidities. In particular, the addition of a malabsorptive component following RYGB has allowed us to treat diabetes in the majority of our patients. This can also be explained by the fact that many of these patients had a rather recent onset of diabetes, often following weight regain after LSG [9,31]. 

Moreover, we also found a better %TWL in patients who experienced WR than in those who had IWL after LSG (Figure 2). These results are consistent with those of other clinical studies, and are in our opinion attributable to multiple factors [32]. Primarily, patients who have experienced WR are often patients who obtain satisfactory results with restrictive techniques but fail for various reasons to maintain the result.

In this case, the conversion to a technique such as RYGB, having a strong restrictive component, allows them once again to obtain good results. On the other hand, patients who experienced IWL, sometimes despite having undergone several restrictive surgeries, are probably patients who have been misdirected to restrictive surgery, and who would probably had better weight loss results if they had undergone an early malabsorptive surgery. 

Therefore, WR after failed bariatric surgery is a positive prognostic factor for the long-term outcomes of RYGB, as shown by the logistic analysis of our study. The need for malabsorptive–metabolic surgery for these patients is also supported by the results obtained with purely restrictive interventions, which are significantly lower than these procedures [29]. Therefore, in our opinion, it is mandatory to perform RYGB in all those patients who show WR after several restrictive procedures.

The significant reduction in the %TWL or %EWL trend after 12 months from a malabsorptive revisional surgery present in the literature [9,20,21,22,23] was the reason for the choice of the cut-off. The studies of these authors showed %EWL at 12 months and at 24 months of 47.4–74%, respectively.

This decrease, comparable to ours, is indicative of the weight reduction potential of RYGB after failed bariatric surgery. The mean value found at 12 months can therefore provide a good indicator of the long-term probability of success of the revisional surgery [8]. The statistical analysis of the regression model confirms this evidence from the literature and the cut-off choice.

In our opinion, the further weight loss recorded after the conversion could be linked to the fact that the changes in ghrelin and GLP-1, induced by LSG, determine a long-term change in the metabolic structure [29]. This hormonal variation could sum up to that induced by the bypass in the medium term advocated by Maleckas et al. (foregut hypothesis) [31]. This could further reduce the caloric intake after malabsorptive surgery. 

From a surgical point of view, a previous history of multiple restrictive operations does not appear to increase the rate of postoperative complications. In fact, the postoperative results show two statistically homogeneous populations regarding the percentage of early and late complications. This is probably due to the fact that in the MRP group, the operation potentially most at risk is the LSG after banding and not the conversion to RYGB. During the LGS, the section of the stomach at the angle of His is performed on a more fragile scar tissue, the site of the previous band. Instead, during the conversion to RYGB, performed on average 4 years later, the transverse section of the stomach to create the gastric pouch is performed far from the site of the previous band. Moreover, all the previous operations before conversion to RYGB were performed laparoscopically and without the necessity of bowel manipulation, and consequently with a low risk of postoperative adhesions. 

As the time interval between the two restrictive measures in the MRP group was very different (0–190 months), it is not possible to attribute significance to this parameter. The removal of LAGB in one-stage surgery (35 patients) was significantly higher in this group than in the two-stage group (9 patients). We prefer the first approach, if possible, as it reduces costs and the number of procedures. However, as shown in the literature, the complication rate in one stage is significantly higher than in two stages due to adhesions, neo-angiogenesis, and the risk of visceral lesions [33].

We believe that in the conversion of LSG to RYGB, the removal of residual antrum and the neo-fundus of the stomach, as some authors point out [26], determine further improvements in hormonal order with implementation of peripheral insulin sensitivity. These data support our findings in relation to DM2. The non-significant difference between the two groups reflects the common anatomical end result of the removal of the two parts of the stomach.

The differences recorded between the last LSG and RYGB timing in the subjects who experienced better results (%EWL) were not significant (*p* = 0.556), and the regression model showed only for the MRP group an association with the time elapsed before RYGB. The results obtained highlighted a linear increase of %EWL with the increase of timing. These results were not found for patients undergoing LSG alone. There are no exhaustive data in the literature on this; however, we believe that this evidence is due to the fact that patients who have failed multiple procedures are much more compliant with post-intervention therapies and indications. Although there is no evidence for this in the current literature, these data could provide a starting point for future studies on the hormonal changes induced by RYGB in a metabolism already altered by restrictive surgery.

In our opinion, these results underline the applicability of the RYGB in the treatment of failed bariatric surgery regardless of the timing since the last surgery. In this sense, future findings could determine the best time to perform revision surgery.

In addition to the limitations related to its retrospective nature, the present study has other limitations due to the small number of patients enrolled, and the fact that, being multicenter, there was no standardization on the indications for surgical revision. This bias arises from the lack of guidelines in this regard, so future randomized prospective studies on larger samples are necessary to confirm the results presented by our study. 

## 5. Conclusions

In conclusion, RYGB proved to be a reliable technique with good results in terms of weight loss after failed bariatric surgery both in patients who had previously undergone MRP and in those who underwent exclusively LSG. RYGB showed better results in patients who experienced WR than in those who had IWL from previous techniques.

## Figures and Tables

**Table 1:** Patient characteristics.

	Total n = 113	MRP n = 44 (38.94%)	LSG n = 69 (61.06%)	*p*-Value
Age, mean ± SD	43.50 ± 10.95	45.73 ± 10.70	42.07 ± 10.95	0.084 *
Sex, n (%)				0.674 **
Female	101 (89.38)	40 (90.91)	61 (88.41)	
Male	12 (10.62)	4 (9.09)	8 (11.59)	
BMI before LSG, mean ± SD	46.08 ± 6.47	45.36 ± 6.95	46.53 ± 6.16	0.351 *
BMI before RYGB, mean ± SD	40.71 ± 5.32	41.53 ± 6.25	40.19 ± 4.60	0.196 *
MRP/LSG–RYGB timing, mean ± SD	49.62 ± 28.88	48.91 ± 24.25	50.07 ± 31.64	0.836 *
Type 2 diabetes	11/9.7%	6/13.6%	5/7.2%	0.33
OSAS	32/28.%	1431.8%	18/26.%	0.52
Hypertension	22/19.5%	10/22.7%	12/17.4%	0.64
%EWL before RYGB	7.22 ± 52.32	22.40 ± 73.72	25.67 ± 14.56	<0.001
Weight condition before RYGB, n (%)				
IWL	99 (87.61)	30 (68.18)	69 (100.00)	<0.001 **
%TWL-24M		21.7 ± 1.1	23.8 ± 9.4	0.163 *
WR	14 (12.39)	14 (31.82)	0 (0.00)	<0.001 **
%TWL-24M		29.4 ± 1.4		
%EWL after RYGB (3M), mean ± SD	29.34 ± 17.07	28.94 ± 14.87	29.59 ± 18.44	0.844 *
%EWL after RYGB (6M), mean ± SD	43.98 ± 20.81	42.76 ± 21.46	44.75 ± 20.51	0.623 *
%EWL after RYGB (12M), mean ± SD	57.66 ± 24.93	55.54 ± 27.37	59.01 ± 23.35	0.474 *
%EWL after RYGB (24M), mean ± SD	62.73 ± 25.13	63.07 ± 26.10	62.51 ± 24.68	0.909 *
%TWL after RYGB (3M), mean ± SD	11.00 ± 6.03	11.03 ± 6.26	10.97 ± 5.92	0.958 *
%TWL after RYGB (6M), mean ± SD	16.40 ± 8.08	16.39 ± 9.11	16.40 ± 7.42	0.995 *
%TWL after RYGB (12M), mean ± SD	21.30 ± 9.43	21.43 ± 11.56	21.22 ± 7.87	0.912 *
%TWL after RYGB (24M), mean ± SD	23.30 ± 9.91	24.22 ± 11.31	22.71 ± 8.93	0.909 *

BMI: body mass index, LSG: laparoscopic sleeve gastrectomy, MRP: multiple restrictive procedures, RYGB: Roux-en-Y bypass, IWL: insufficient weight loss, WR: weight regain, %EWL: % excess weight loss, %TWL: % total weight loss, OSAS: obstructive sleep apnea syndrome; * Student’s *t*-test, ** Fisher’s exact test.

**Table 2:** Linear regression between %EWL after RYGB (24M), MRP/LSG–RYGB timing and %EWL before RYGB in the groups.

	Linear Regression		
	β	95% CI	*p*-Value
MRP group			
MRP–RYGB timing	0.53	0.23–0.82	0.001
%EWL before RYGB	−0.12	−0.22–−0.01	0.031
LSG group			
LSG–RYGB timing	0.08	−0.11–0.27	0.404
%EWL before RYGB	0.18	−0.23–0.59	0.378

%EWL: % excess weight loss, RYGB: Roux-en-Y bypass, LSG: laparoscopic sleeve gastrectomy, MRP: multiple restrictive procedures.

**Table 3:** Univariate logistic model.

	%EWL after RYGB (24M)		Univariate Logistic Model		
	>MV-12M n (%) 88 (77.88)	≤MV-12M n (%) 25 (22.12)	OR	95% CI	*p*-Value
Age, mean ± SD	44.26 ± 10.87	40.80 ± 11.04	0.97	0.93–1.01	0.165
Sex, n (%)					
Female	78 (88.64)	23 (92.00)	1		
Male	10 (11.36)	2 (8.00)	0.68	0.14–3.32	0.632
BMI LSG, mean ± SD	46.47 ± 6.85	44.68 ± 4.77	0.95	0.88–1.03	0.222
Duration LSG, mean ± SD	48.76 ± 28.91	52.64 ± 29.15	1.00	0.99–1.02	0.553
BMI, RYGB mean ± SD	40.93 ± 5.37	39.94 ± 5.12	0.96	0.88–1.05	0.408
MRP n (%)	39 (44.32)	5 (20.00)	1		
LSG n (%)	49 (55.68)	20 (80.00)	3.18	1.10–9.25	0.033

%EWL: % excess weight loss, RYGB: Roux-en-Y bypass, LSG: laparoscopic sleeve gastrectomy, MRP: multiple restrictive procedures.

**Table 4:** Comorbidities’ responses to surgical treatment.

	MRP	LSG	*p*-Value	MRP	LSG	*p*-Value	MRP	LSG	*p*-Value
	I	I		R	R		S	S	
T2D	4/6	3/5	1	/	/	/	2/6	2/5	0.56
AHT	5/10	2/12	0.17	3/10	6/12	0.41	2/10/	4/12	0.64
OSAS	14/14	16/18	1	/	/	/	2/14	2/18	1

MRP: multiple restrictive procedures; LSG: laparoscopic sleeve gastrectomy; T2D: type 2 diabetes; AHT: arterial hypertension; OSAS: obstructive sleep apnea syndrome. I: improved; R: resolved; S: stable.

## Data Availability

The datasets generated during and/or analyzed during the current study are available from the corresponding author on reasonable request.
